# Genetic Structure and Molecular Variability Analysis of *Citrus sudden death-associated virus* Isolates from Infected Plants Grown in Brazil

**DOI:** 10.3390/v8120330

**Published:** 2016-12-16

**Authors:** Emilyn Emy Matsumura, Helvécio Della Coletta Filho, Silvia de Oliveira Dorta, Shahideh Nouri, Marcos Antonio Machado

**Affiliations:** 1 Instituto de Biociências de Botucatu, Universidade Estadual Paulista, Botucatu, São Paulo 03178-200, Brazil; emilyn.matsumura@gmail.com; 2 Laboratório de Biotecnologia, Centro de Citricultura Sylvio Moreira, Instituto Agronômico, Cordeiropolis, SP 13490-970, Brazil; helvecio@centrodecitricultura.br (H.D.C.F.); dorta.silvia@gmail.com (S.d.O.D.); 3 Department of Plant Pathology, University of California Davis, Davis, CA 95616, USA; shahidehnr@gmail.com

**Keywords:** citrus sudden death, CSDaV, plant virus, *Marafivirus*, diversity

## Abstract

*Citrus sudden death-associated virus* (CSDaV) is a monopartite positive-sense single-stranded RNA virus that was suggested to be associated with citrus sudden death (CSD) disease in Brazil. Here, we report the first study of the genetic structure and molecular variability among 31 CSDaV isolates collected from both symptomatic and asymptomatic trees in CSD-affected areas. Analyses of partial nucleotide sequences of five domains of the CSDaV genomic RNA, including those encoding for the methyltransferase, the multi-domain region (MDR), the helicase, the RNA-dependent RNA polymerase and the coat protein, showed that the MDR coding region was the most diverse region assessed here, and a possible association between this region and virus adaption to different host or plant tissues is considered. Overall, the nucleotide diversity (π) was low for CSDaV isolates, but the phylogenetic analyses revealed the predominance of two main groups, one of which showed a higher association with CSD-symptomatic plants. Isolates obtained from CSD-symptomatic plants, compared to those obtained from asymptomatic plants, showed higher nucleotide diversity, nonsynonymous and synonymous substitution rates and number of amino acid changes on the coding regions located closer to the 5’ end region of the genomic RNA. This work provides new insights into the genetic diversity of the CSDaV, giving support for further epidemiological studies.

## 1. Introduction

*Citrus sudden death-associated virus* (CSDaV) is a member of the genus *Marafivirus* in the family *Tymoviridae*, and has shown a strong association with Citrus sudden death (CSD), an important citrus disease in Brazil [1]. CSDaV virions are isometric particles of ≈30 nm in diameter, composed of a monopartite, positive-sense, single-stranded RNA genome of approximately 6.8 kb with a high cytosine content (37.4%) and encompassing two ORFs [1,2]. A large ORF (ORF1) encodes a 240 kDa polyprotein (p240) which contains conserved signatures of domains involved with replication and virion structure, including the methyltransferase (MT), the papain-like protease (PRO), the helicase (He), the RNA-dependent RNA polymerase (RdRP) domains and two subunits of the coat protein (CP) of 21 and 22 kDa in size, respectively [1]. Moreover, a multi-domain region that contains numerous predicted single domains is detected in ORF1 (between the MT and PRO domains), but the function of this region in CSDaV is unknown. The small ORF (ORF 2) at the 3’ end region encodes a 16 kDa putative protein (p16) that has shown 42% identity with the N-terminal portion of a putative movement protein (p31) from *Grapevine fleck virus* (GFkV), a member of the genus *Maculavirus* in the family *Tymoviridae* [1].

The first report of CSD was in 1999, affecting sweet oranges (*Citrus sinensis* L. Osb.) grafted on Rangpur lime rootstock (*Citrus limonia* L. Osb.), the main non-irrigated rootstock used in Brazil [3]. Since then, CSD has caused death or eradication of four million orange trees in Minas Gerais and São Paulo states [4]. Recently, CSD-symptoms have been also detected in sweet oranges grafted on other rootstocks (e.g., *Citrus volkameriana*, *Citrus jambiri* and *Citrus pennivisiculata* Lush) [5]. Citrus plants affected by CSD show general decline symptoms characterized by pale green coloration of leaves, different levels of defoliation, death of the root system, and a characteristic development of yellow stain in the phloem of the rootstock [6], which is the main diagnostic symptom of this disease [3,6]. However, these affected plants had an incubation period of at least 2 years before symptoms were detected [1,6], which may result in delay of management of the disease. Although the etiology of CSD has not been definitively determined, Maccheroni et al. [1] reported a significant correlation at 99.7% between CSD symptoms and the presence of CSDaV, and suggested that it is probably spread by an aphid vector. The presence of CSDaV as a part of a multiple virus infections or co-infections has been reported in other hosts as well, such as in Pinot Noir grapevine [7], in Nectarine [8] and in grapevine Syrah showing decline symptoms [9]. Such co-infections are also considered for plants showing CSD symptoms [1,4,10].

Only two CSDaV isolates have been characterized so far, and their complete genome sequences are available in GenBank (accession No. AY884005 and DQ185573). However, the structure of CSDaV populations has not been studied and the evolutionary forces that may shape this structure are still unknown. To better understand the relationship between CSDaV and CSD, we studied the genetic structure and molecular variability among CSDaV isolates obtained from CSD-affected areas, and compared them with reference isolates by analyzing the partial nucleotide sequences of five coding regions including those for MT, the multi-domain region (named here as MDR), the He, the RdRP and the CP. As a result, we showed that the MDR region was the most diverse region assessed here. We identified the predominance of two main phylogenetic groups, one of which showed a higher association with CSD-symptomatic plants. CSDaV isolates from CSD-symptomatic plants showed higher nucleotide diversity, nonsynonymous and synonymous substitution rates and number of amino acid changes on the coding regions located closer to the 5’ end region of the genomic RNA. These results provide relevant information for further epidemiological studies.

## 2. Materials and Methods

### 2.1. Plant Collection

The CSDaV population was assessed from different citrus plants: different cultivars of sweet orange grafted on different rootstocks, susceptible and tolerant to CSD. A total of 31 plants was sampled: fifteen trees were asymptomatic and 16 trees had clear CSD symptoms (i.e., occurrence of yellow stain in the rootstock bark), including a tree grafted on Sunki mandarin of China, which is supposed to be tolerant to CSD, and trees grafted on CSD-susceptible rootstock (Rangpur lime), but intergrafted with tolerant rootstocks (Trifoliate orange and Cleopatra mandarin). Genotypes and symptom information are summarized in Table 1. All selected trees were monitored since 2003 in CSD-affected areas located in the municipalities of Colombia (northern Sao Paulo State) and Comendador Gomes (southwestern Minas Gerais state), Brazil. CSD-symptomatic plants showed the first symptoms in 2006. All citrus plants were approximately five years old at the time of collection in 2007. Collected samples were frozen in liquid nitrogen and stored at −80 °C prior to analysis.

### 2.2. RNA Extraction and RT-PCR Amplification

**Figure 1:**
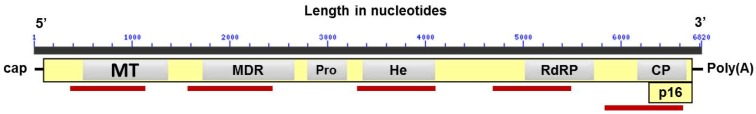
Genome organization of CSDaV. The two ORFs are represented by yellow boxes and the conserved domains are represented by grey boxes. Red bars indicate the genomic regions analyzed in this study.

Total RNA was extracted from all samples using the RNeasy Plant Mini kit (Qiagen, Valencia, CA, USA) according to the manufacturer’s instructions. A set of primers (Table 2) was designed to amplify nucleotide sequences, which corresponded partially to the five domains: the methyltransferase (MT), the multi-domain region (MDR), the helicase (He), the RNA-dependent RNA polymerase (RdRP) and the coat protein (CP) coding regions based on the CSDaV reference genomes (GenBank accession no. AY884005 and DQ185573) (Figure 1). cDNAs were synthesized in a 20 µL volume of 1× Reaction Buffer, containing 0.5 mM dNTPs mix, 200 U of RevertAid H Minus M-MuLV Reverse Transcriptase (Thermo Scientific, Waltham, MA, USA), and 5 µM of a random hexamer primer. PCR reactions were performed in 25 µL volume, containing 1× High Fidelity PCR Buffer (Invitrogen, Carlsbad, CA, USA), 0.2 mM dNTP mix, 2 mM MgSO4, 0.02 U of Platinum Taq DNA Polymerase High Fidelity (Invitrogen) and 10 mM of each forward and reverse primers. The following PCR conditions were used: 94 °C for 2 min; 35 cycles each of 94 °C for 15 s, 55 °C (for all pair of primers) for 30 s and 68 °C for 1 min. The resulted PCR products were separated by electrophoresis in a 1% agarose gel and detected by ethidium bromide staining. Bands were cut from the gel and the PCR products were purified using a QIAquick gel extraction kit (Qiagen).

### 2.3. Cloning and Sequencing

The purified PCR products were cloned into pGEM-T vector (Promega, Madison, WI, USA) using T4 DNA ligase (Promega) according to the manufacturer’s instructions, followed by transformation into *Escherichia coli* DH5α competent cells [11]. Ten recombinant colonies were selected on screening media and confirmed by colony PCRs. Plasmid DNAs were extracted using the PureYield plasmid miniprep system kit (Promega) following the manufacturer’s instructions and were bi-directionally sequenced using an Applied Biosystems 3730 DNA Analyzer.

### 2.4. Nucleotide Sequence Analysis

CSDaV reference sequences, identified as AY884005 (CSDaV) and DQ185573 (CSDaV strain p15) were downloaded from GenBank [12] and included in this analysis as representatives of CSDaV. Multiple nucleotide sequence alignments for each genomic region were obtained using the CLUSTAL W [13], and manually edited in the program MEGA 6.06 [14]. Neighbor joining phylogenetic trees were inferred with 1000 bootstraps in the MEGA 6.06 program and the generated trees were edited using FigTree version 1.4.2 [15]. A set of sequences for each genomic region of CSDaV were assessed using DnaSP software version 5.1 [16] to estimate genetic diversity and other population genetic parameters.

Recombination events among CSDaV isolates were examined using phylogenetic analysis and the boot-scan method in the SimPlot program [17]. Evidence of recombination was considered when 70% of permuted trees supported a particular grouping of sequences. The window width and the step size were set to 200 and 20 bp, respectively. The degree of selective constraints imposed on different regions of CSDaV genome was estimated with MEGA 6.06 program by analyzing the nonsynonymous and synonymous substitutions ratios (dN/dS = ω) using the Kumar method and bootstrap with 500 replicates [18]. Fixed effects likelihood (FEL), random effects likelihood (REL), and single likelihood ancestor counting (SLAC) tests, all included in the Hyphy package [19], were performed to determine the site specific selection pressure for the coding regions. For SLAC and FEL, the cut-off *p*-value was defined as 0.1 and for REL, a Bayes factor of 50 was selected as the cut-off value. Only positive selections determined by at least two methods were accepted [20].

## 3. Results

### 3.1. Genetic Diversity of CSDaV Population

The presence of CSDaV was confirmed immediately after plants collection in both symptomatic and asymptomatic plants, including trees grafted on the CSD tolerant rootstocks, such as Cleopatra and Sunki mandarins, Swingle citrumelo and *Poncirus trifoliata*. Two step RT-PCRs with specific degenerate primers sets (Table 2) generated amplicons with 762, 827, 806, 797, 730 bp in length for the five regions of CSDaV genomic RNA including those encoding for the MT, the MDR, the He, the RdRp and the CP, respectively [21]. Figures S1 and S2 and Tables S1 and S2 in the supplemental material show all conserved domains detected from conserved domain search using the CSDaV reference genomes as queries in the NCBI Conserved Domain Database (CDD). A total of 31 CSDaV isolates were obtained (Table 3) and the number of sequences for each region is illustrated in Table 4. Nucleotide diversities were estimated based on the number of segregating sites (θw) and the average number of nucleotide differences per site between sequences (π). Overall, the genetic diversity for CSDaV isolates evaluated in this study was low ranging from 0.01013 (the CP fragment) to 0.04185 (the He fragment) (Table 4) with a mean genetic diversity of 0.026118.

### 3.2. Phylogenetic Relationships of CSDaV Isolates

**Figure 2:**
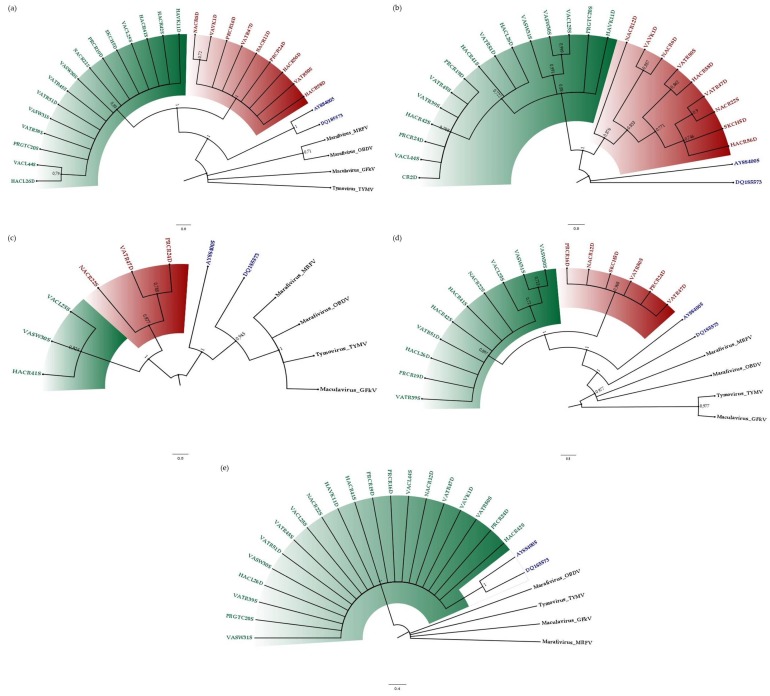
Bootstrap majority rule (70%) consensus trees reconstructed by the neighbor joining method for five genomic regions of CSDaV isolates including field collected and reference sequences. Bootstrap support values (1000 iterations) of main branches are indicated. (**a**) MT segment; (**b**) MDR segment; (**c**) He segment; (**d**) RdRP segment; (**e**) CP segment. CSDaV groups are highlighted by different colors: Group I = green; Group II = red. The CSDaV reference isolates are represented in blue. The outgroups are represented in black. Isolates from asymptomatic plants are identified by letter S at the end of their identification names. Isolates from symptomatic plants are identified by letter D at the end of their identification names.

The sequences of four representative viruses from the genera *Tymovirus* (*Turnip yellow mosaic vírus*—TYMV, NC_004063), *Maculavirus* (*Grapevine fleck virus*—GFkV, NC_003347) and *Marafivirus* (*Maize rayado fino virus*—MRFV, NC_002786; and *Oat blue dwarf vírus*—OBDV, NC_001793) of the family *Tymoviridae* were obtained from GenBank and used as outgroups in the phylogenetic analysis of all regions except the MDR segment because this region of CSDaV did not show any homology with any genomic region of four *Tymoviridae* representatives. Because six isolates were detected as possible recombinants based on the topology of phylogenetic trees (see details in the recombination analysis section), the final trees were constructed after removing these recombinants. In general, the topology of the MT (Figure 2a), the MDR (Figure 2b), the He (Figure 2c) and the RdRP (Figure 2d) trees was similar and showed the presence of two main groups of CSDaV isolates assessed in this study with high supporting bootstrap values equal or higher than 83%. The intra-group diversity was best illustrated in the MDR tree (Figure 2b). The topology of the CP tree was different, in which all CSDaV isolates formed a single un-resolved polytomy clade with a supporting bootstrap value of 99% (Figure 2e). For all phylogenetic trees, with the exception of the CP, the main groups were clustered separately from the two CSDaV reference sequences. Divergence between CSDaV reference sequences (AY884005 and DQ185573 isolates) was observed in the MDR, He and RdRP trees (Figure 2b–d).

### 3.3. Comparison of Genetic Diversity between Isolates from Asymptomatic and Symptomatic Plants

Based on the MT, the MDR, the He and the RdRP phylogenetic trees, group I of the CSDaV isolates was formed by the majority of isolates from asymptomatic plants, whereas group II contained the majority of isolates from symptomatic plants (Figure 2 and Table 5). To further strengthen these results, CSDaV consensus sequences were obtained from transcriptome sequencing, conducted for both symptomatic and asymptomatic plants by using Illumina next generation sequencing (NGS) technology [22]. The coding regions studied here were accessed in these consensus sequences and included in the phylogenetic analysis. Based on the MT, the MDR, the He and the RdRP, the consensus sequence obtained from the asymptomatic library clustered close to the reference isolates, whereas the consensus sequence obtained from the symptomatic library was grouped in group II (Figure S3), which strongly supports the results presented above.

Compared to isolates from asymptomatic plants, the nucleotide diversities estimated only for isolates obtained from symptomatic plants were higher at about 2.2, 1.5, 1.1, 1.1 and 0.9 times for the MT, MDR, He, RdRP and CP regions, respectively (Table 6). The dN/dS ratio values were higher for the MDR region for isolates from both symptomatic and asymptomatic plants. However, this estimated value for the isolates from symptomatic plants was 1.4 times higher than the ratio estimated for the isolates from asymptomatic plants (Table 6). The deduced amino acid sequences from each CSDaV genomic region showed silent mutations between isolates from symptomatic and asymptomatic plants (Table 7).

### 3.4. Recombination Analysis

**Figure 3:**
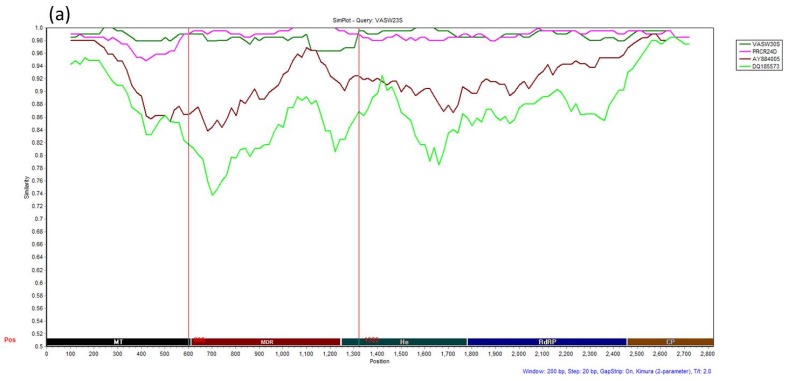

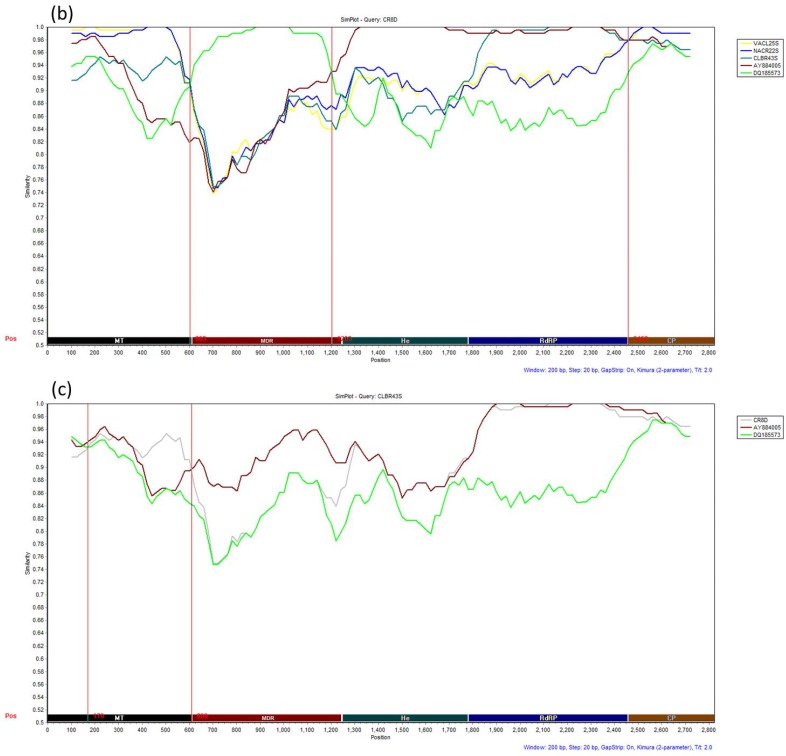
Phylogenetic relationship with potential recombinant CSDaV isolates as the query sequences based on concatenated nucleotide sequences of the MT, MDR, He, RdRP and CP genomic regions using Simplot. Three CSDaV isolates, VASW23S (**a**), CR8D (**b**) and CLBR43S (**c**), were used as query sequences and two CSDaV isolates were used as reference sequences. The Y-axis illustrates variation in identity percentage. Analyses were done using a sliding window of 200 bp and a step size of 20 bp. Red vertical dashed line shows the proposed recombination break point. Sequences compared with the query sequence are indicated in the legend.

**Figure 4:**
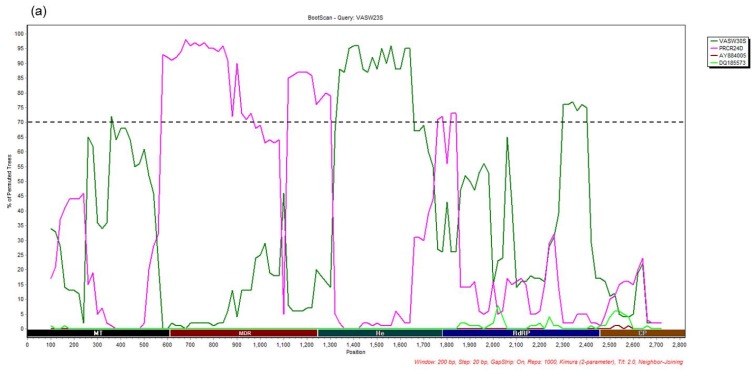

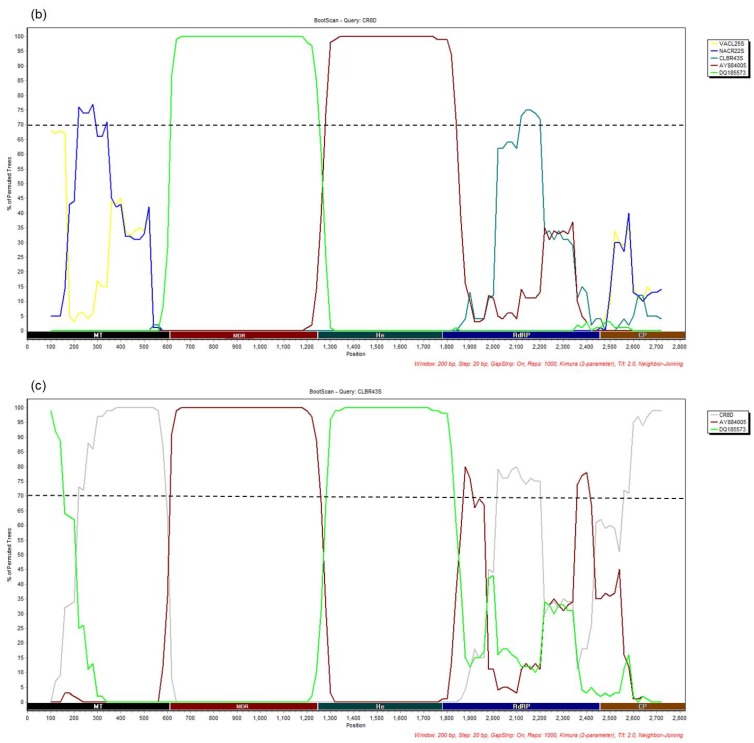
Bootscan analyses with potential recombinant CSDaV isolates as the query sequences based on concatenated nucleotide sequences of the MT, MDR, He, RdRP and CP genomic regions using Simplot. Three CSDaV isolates, VASW23S (**a**), CR8D (**b**) and CLBR43S (**c**), were used as query sequences and two CSDaV isolates were used as reference sequences. The Y-axis illustrates variation in percentage of permuted trees in which each selected isolate clustered with the query sequence. Analyses were done using a sliding window of 200 bp and a step size of 20 bp. Black dashed line shows the 70% cutoff level, representing possible recombination. Sequences compared with the query sequence are indicated in the legend.

Based on the phylogenetic trees constructed with all, including the possible recombinants CSDaV isolates (Figure S4), six isolates, named CR8D, CLBR43S, VASW23S, HACL38S, HACL52D and HACR55S showed some phylogenetic incompatibilities and evidence of recombination events. The two root isolates (CLBR43S and CR8D) clustered in the same clade according to the RdRP and the CP phylogenetic trees (Figure S4d,e), while they were placed separately based on the MT, the MDR and the He trees (Figure S4a–c). Isolate VASW23S grouped separately in MT and the RdRP phylogenetic trees (Figure S4a,d), but it clustered with the main groups in the MDR, the He and the CP trees (Figure S4b,c,e). Isolates HACL38S, HACL52D and HACR55S were not compared in all regions of the genome analyzed here because we were not able to obtain PCR products for all segments, and they were excluded from the recombination analysis. We selected nine representative isolates from this study including three suggested recombinants and six isolates representing the two main groups of CSDaV, and two CSDaV reference sequences to concatenate their nucleotide sequences of the MT, the MDR, the He, the RdRP and the CP segments. Concatenated sequences were further analyzed using SimPlot. Both phylogenetic and Bootscan methods included in Simplot identified recombination signals as well as their possible parental sequences when VASW23S, CR8D and CLBR43S isolates were used as queries (Figure 3 and Figure 4). Phylogenetic analysis of the concatenated sequences detected several recombination hotspots when different isolates were used as queries: positions 600 and 1322 when VASW23S isolate was used as the query (Figure 3a), positions 603, 1203 and 2458 when CR8D isolate was used as the query (Figure 3b) and positions 170 and 609 when CLBR43S isolate was used as the query (Figure 3c). On the other hand, Bootscan analyses demonstrated that the MDR and He segments of VASW23S isolate come from PRCR24D-like and VASW30S-like isolates, respectively (Figure 4a). For the CR8D isolate, a recombination event was detected by the Bootscan algorithm in which the MDR and He segments of CR8D were generated from two different origins: AY884005 and DQ185573 reference isolates, respectively (Figure 4b). When Bootscan analysis was performed using CLBR43S isolate as the query, we detected four recombination hotspots in which two of them were placed close to positions 170 and 619 (already shown by phylogenetic analysis), and two other hotspots were detected at positions 1271 and 1850 (Figure 3c and Figure 4c). Furthermore, Bootscan results confirmed that the MDR and He segments of CLBR43S were generated from two CSDaV reference isolates (Figure 3c) and the region from the MT segment was likely driven by recombination events between a DQ185573 reference-like isolate and CR8D-like isolate. The RdRP and the CP segments from CLBR43S isolate showed phylogenetically inconsistent regions with some similarity with the CR8D isolate and the AY884005 reference isolate (Figure 4c).

Only the two root isolates detected as recombinants showed a close phylogenetic relationship to one of the CSDaV reference isolates: CR8D isolate in the MDR, the He and the RdRP trees and CLBR43S isolate in the RdRP tree (Figure S4). However, these isolates were phylogenetically distant from the main groups of CSDaV isolates assessed in this study. According to the MT, MDR, He and RdRP phylogenetic trees, isolate CLBR43S, obtained from tissues of Cleopatra mandarin rootstock, was found in a separate clade which was phylogenetically distant from the two main groups of CSDaV isolates (Figure S4). Similar results were found for isolate CR8D, obtained from a Rangpur lime rootstock, according to MDR, He and RdRP phylogenetic trees (Figure S4). Both of those separations were well-supported.

### 3.5. Selective Pressure for Different Genomic Regions of CSDaV

Evidence of positive selection was not found in any region of the genome for the CSDaV isolates included in this study. The mean ω (dN/dS) value for all genomic regions analyzed here was less than 1.0, indicating that all segments were subjected to negative or purifying selection. Among regions, the MT, He, RdRP and CP regions showed low ω ratios, while this ratio was higher for the MDR region (Table 4). Moreover, complementary maximum-likelihood methods (SLAC, FEL and REL) detected positively selected sites only for the MDR segment. Site 20 in the MDR segment was considered to be significant under positive selection by two methods: FEL (dN − dS = 19.1017 and *p*-value = 0.042) and REL methods (dN − dS = 5.2268 and Bayes Factor = 23853.7) (A→T).

## 4. Discussion

We provided for the first time a snapshot of the genetic structure and variability among Brazilian CSDaV isolates collected from both symptomatic and asymptomatic citrus trees grown in fields affected by CSD disease. To date, only two CSDaV genome sequences were fully described, showing 11% nucleotide diversity between them [23] (GenBank accession no. AY884005 and DQ185573). Both of these well-described CSDaV isolates were obtained from Rangpur lime tissues as rootstock of sweet orange trees collected from the same citrus region assessed in this study [1,24], which is relevant since we can study the evolution of this virus in this CSD-affected area. In the current study, sequence analyses of five regions of the CSDaV genome representing almost 42% of the whole genome of 31 isolates, sampled from different hosts/plant tissues, showed a low genetic diversity. It is not a surprising finding because genetic stability has been considered as a rule in natural plant virus populations [23] and similar low genetic diversity was previously reported for many other RNA plant viruses [20,25,26,27,28,29,30,31]. It has been shown that systemic infections and other events such as host change and transmission can impose bottlenecks, the most common effects of genetic drift, which have been inferred from the low genetic diversity of plant virus populations [32,33] and which might be the reason for the low genetic diversity among the CSDaV isolates.

The low nucleotide variability observed for the CP, the MT and the RdRP regions of CSDaV genome included in this study suggests that selective pressures in these segments are high to maintain nucleotide and amino acid conservation probably for biological functions [34]. It has been shown that the coat protein (CP) plays critical roles in virus packaging and stability, and interactions with plant host [34]. Similarly, the MT and the RdRP domains play key roles in viral replication, involving mRNA capping and enhanced stability of viral genomes (methyltransferase) [35]; and transcription and replication of RNA virus genomes (RdRP) [36]. On the other hand, the MDR and He regions demonstrated higher nucleotide variability. The MDR segment showed the highest genetic diversity among all studied regions here, and it was the only region that had one site detected as being under positive selection. The MDR segment represents a multi-domain region that contains numerous predicted single domains related to different activities. Interestingly, the MDR was the unique multi-domain region found along the CSDaV genome and was the single region that we could not align with other reference members of the family *Tymoviridae*. It seems that this region is unique and associated with CSDaV isolates and could be related with some processes of virus adaption [37] to a different host or plant tissues. However, at this time, there is no information about the function(s) of this multi-domain, and then further studies are needed to evaluate the real role of this region in CSDaV. Probably because the pair of primers designed for the He region was highly degenerate, we were not able to amplify the He segment in several samples assessed here and it may be possible that the low number of isolates (nine) has influenced the results. From this work, it is clear that there is a genetic diversity between the CSDaV isolates assessed here and the CSDaV references previously reported. The only isolates that showed close phylogenetic relationships with the CSDaV reference isolates were those isolated from the citrus roots, which were also detected as recombinants in this work, pointing the CSDaV reference sequences as the possible parents. Since we know that the CSDaV reference isolates were isolated from rootstock tissues of citrus trees as well [1,24], these results also provide some evidence of the heterogeneous distribution of virus variants at different locations (leaves and roots) within hosts. Other studies have already reported that the diversity of virus populations is different between old and young tissues, suggesting the tree could reflect the chronology of the appearance of virus diversity [38].

Phylogenetic analyses showed two new genetic clades for the CSDaV isolates included in this investigation, and one of them showed higher association with symptomatic trees. Higher nucleotide diversity, dN/dS ratio values and number of amino acid changes were found for isolates from symptomatic plants in coding regions located closer to the 5’ end region of the CSDaV genome (MT and MDR), whereas coding regions located closer to the 3’ end region showed more conservation. It is important to say that these isolates belonging to these two new genetic clades were all isolated from the citrus leaves, which have been shown to have CSDaV variants, compared to the CSDaV isolates from the roots (this work and the references isolates). It is possible that the CSDaV isolates infecting rootstock tissues were subjected to some positive selection pressures, mainly on the coding regions closer to the 5’ end region, to be able to infect tissues in the citrus canopy, culminating with two different variants of CSDaV, where one of them might be more efficient in infecting CSD-susceptible plants and/or more severe in developing CSD symptoms. Other factors, such as the susceptibility of the citrus rootstock and climate (drought and higher temperature), seem to contribute to the development of the CSD. The confirmed presence of CSDaV in trees grafted on symptomatic and asymptomatic susceptible rootstocks, and symptomatic and asymptomatic tolerant rootstocks, suggest that CSDaV is able to infect a wide host range in CSD-affected regions, but the symptoms are not always developed. The results obtained here do not discard the possibility of a mixed or co-infection of the CSDaV and other virus(es), which was already proposed as a cause of CSD [1]. CSDaV and other members of the genus *Marafivirus* have been frequently associated in mixed or co-infections in other pathosystems. CSDaV was found to be part of a multiple virus infection in Pinot Noir grapevine [7] and in grapevine Syrah showing decline symptoms [9]. Recently, Villamor et al. [8] found CSDaV infecting California nectarines showing stem-pitting symptoms and also revealed the presence of a new virus of the genus *Marafivirus*, which shared 70% of nucleotide sequence similarities to CSDaV, co-infecting these plants. All these results obtained in this investigation could together provide new insights into the role of CSDaV in symptom development in plants affected by CSD and contribute to further epidemiological studies.

## Figures and Tables

**Table 1:** Citrus plants used to assess the population of CSDaV. Canopy and rootstock genotypes of each plant are shown. Type of plant tissue and number of collected plants are indicated.

	Canopy (*C. sinensis*)	Rootstock	Collected Tissue	Number of Plants
**Asymptomatic plants**	Natal	Rangpur lime (*C. limonia*)	Leaves	1
	Valencia	Swingle citrumelo (*P. trifoliate* x C. paradisi)	Leaves	3
	Hamlin	Rangpur lime (*C. limonia*)	Leaves	3
	Pera Rio	Gou Tou (unidentified *Citrus* hybrid)	Leaves	1
	Valencia	Cleopatra mandarin (*C. reshni*)	Leaves	2
	Valencia	Trifoliate orange (*P. trifoliata*)	Leaves	3
	Hamlin	Cleopatra mandarin (*C. reshni*)	Leaves	1
	Hamlin	Cleopatra mandarin (*C. reshni*)	Roots	1
**Symptomatic plants**	Valencia	Volkamerian lemon (*C. volkameriana*)	Leaves	1
	Natal	Rangpur lime (*C. limonia*)	Leaves	2
	Hamlin	Rangpur lime (*C. limonia*)	Leaves	2
	Hamlin	Volkamerian lemon (*C. volkameriana*)	Leaves	1
	Valencia	Rangpur lime (*C. limonia*) and Trifoliate orange (*P. trifoliata*) as interstock	Leaves	2
	Pera Rio	Rangpur lime (*C. limonia*)	Leaves	3
	Hamlin	Rangpur lime (*C. limonia*) and Cleopatra mandarin (*C. reshni*) as interstock	Leaves	2
	Hamlin	Rangpur lime (*C. limonia*)	Roots	2
	Valencia	Sunki mandarin of China (*C. sunki*)	Leaves	1
**Total = 31 plants**				

**Table 2:** Primer sequences designed based on five genomic regions of CSDaV genome.

Genomic Region	Primer Sequences (5’–3’)	Annealing Nucleotide Position
**MT**	Forward-CGTCAAACTCCCNCTGAC	351–368
	Reverse-GATCANNAGAGAGTGGACTG	1094–1113
**MDR**	Forward-CTCCCTCTCCATCTGCAAGC	1566–1585
	Reverse-ATANTCNNTGGAGGGGTCA	2375–2393
**He**	Forward-AGATNTTGGCNCTNGANTC	3305–3323
	Reverse-ANTCNGAGAACATTCNGTTG	4092–4111
**RdRP**	Forward-CATCAAGAGAANCANGANCC	4636–4355
	Reverse-TGAGACCATAGTGGGAGTGT	5414–5433
**CP**	Forward-GCCATCTACACCACACTCTC	5857–5876
	Reverse-TTGGANTAGACGGAGTAGGA	6568–6587

**Table 3:** List of CSDaV sequences obtained in this work. Accession numbers in the GenBank database for the different genomic regions of each isolates are indicated.

Isolate Identification *	Viral Genomic Region	GenBank Accession No.
VAVK1D	MT	KX753236
	MDR	KX753259
	CP	KX753328
CR2D	MDR	KX753263
HAVK11D	MT	KX753252
	MDR	KX753260
	CP	KX753326
NACR12D	MT	KX753233
	MDR	KX753261
	RdRP	KX753309
	CP	KX753327
PRCR19D	MT	KX753245
	MDR	KX753262
	RdRP	KX753306
	CP	KX753330
PRGTC20S	MT	KX753254
	MDR	KX753264
	CP	KX753321
VASW23S	MT	KX753248
	MDR	KX753265
	He	KX753296
	RdRP	KX753316
	CP	KX753340
PRCR24D	MT	KX753234
	MDR	KX753266
	He	KX753292
	RdRP	KX753313
	CP	KX753336
HACL26D	MT	KX753243
	MDR	KX753267
	RdRP	KX753307
	CP	KX753323
CR8D ^1^	MT	KX753257
	MDR	KX753268
	He	KX753297
	RdRP	KX753318
	CP	KX753342
VASW30S	MT	KX753256
	MDR	KX753269
	He	KX753293
	RdRP	KX753299
	CP	KX753324
VASW31S	MT	KX753242
	MDR	KX753270
	RdRP	KX753298
	CP	KX753319
HACL38S	MT	KX753244
	MDR	KX753271
	RdRP	KX753301
	CP	KX753320
VATR39S	MT	KX753255
	MDR	KX753272
	RdRP	KX753305
	CP	KX753322
HACR42S	MT	KX753241
	MDR	KX753273
	RdRP	KX753302
	CP	KX753333
CLBR43S ^2^	MT	KX753251
	MDR	KX753274
	He	KX753294
	RdRP	KX753317
	CP	KX753334
VACL44S	MT	KX753247
	MDR	KX753275
	CP	KX753341
VATR45S	MT	KX753239
	MDR	KX753276
	CP	KX753332
VATR47D	MT	KX753253
	MDR	KX753277
	He	KX753291
	RdRP	KX753314
	CP	KX753343
SKCH5D ^3^	MT	KX753237
	MDR	KX753278
	RdRP	KX753310
VATR50S	MT	KX753249
	MDR	KX753279
	RdRP	KX753312
	CP	KX753329
VATR51D	MT	KX753258
	MDR	KX753280
	RdRP	KX753304
	CP	KX753325
HACL52D	MDR	KX753281
	RdRP	KX753315
HACR55S	MDR	KX753282
	CP	KX753331
HACR56D	MT	KX753235
	MDR	KX753283
HACR58D	MT	KX753250
	MDR	KX753284
NACR6D	MT	KX753232
	MDR	KX753285
VACL25S	MT	KX753238
	MDR	KX753286
	He	KX753289
	RdRP	KX753300
	CP	KX753337
NACR22S	MT	KX753246
	MDR	KX753287
	He	KX753295
	RdRP	KX753308
	CP	KX753339
HACR41S	MT	KX753240
	MDR	KX753288
	He	KX753290
	RdRP	KX753303
	CP	KX753338
PRCR16D	MT	KX753231
	RdRP	KX753311
	CP	KX753335

* Isolates were designated based on the citrus genotype from where the CSDaV isolates were obtained. First two letters identify the type of canopy (VA: Valencia; HA: Hamlin; PR: Pera Rio and NA: Natal), followed by the type of rootstock or interstock (VK: Volkameriano lemon; CR: Rangpur lime; GTC: Goutou; SW: Swingle citrumelo; CL: Cleopatra mandarin; TR: Trifoliate orange), the sample number and the symptom information (S: asymptomatic plant and D: symptomatic plant). ^1^ Isolate from Rangpur lime rootstock tissues; ^2^ Isolate from Cleopatra mandarin rootstock tissues; ^3^ Isolate from leaves of Valencia grafted on Sunki mandarin of China.

**Table 4:** Population genetic parameters estimated for five coding regions of CSDaV isolates using the DnaSP and MEGA programs.

Genomic Regions	Number of Final Sequences	S	η	π	θw	dN	dS	ω (dN/dS)
MT	28	82	84	0.01815	0.0346	0.005 ± 0.002	0.054 ± 0.010	0.093
MDR	30	180	214	0.04091	0.07212	0.023 ± 0.004	0.097 ± 0.012	0.237
He	9	81	83	0.04185	0.05613	0.006 ± 0.002	0.153 ± 0.020	0.039
RdRP	21	70	72	0.01955	0.02895	0.001 ± 0.001	0.068 ± 0.009	0.015
CP	25	26	27	0.01013	0.01897	0.003 ± 0.001	0.026 ± 0.007	0.115

S: Total number of segregating sites; η: Total number of mutations; π: Nucleotide diversity, average pairwise nucleotide difference per site; θw: Mutation rate estimated from S; dN: The average number of pairwise differences per synonymous site; dS: The average number of pairwise differences per non-synonymous site. dS and dN were estimated by the Kumar method.

**Table 5:** Number of CSDaV sequences from symptomatic and asymptomatic plants between the two main phylogenetic groups assessed in this study.

	Number of Isolates from Symptomatic Plants/Number of Isolates from Asymptomatic Plants	
	Group I *	Group II **
**MT**	5/10	8/1
**MDR**	6/9	7/2
**He**	0/3	2/1
**RdRP**	3/7	5/1

* Group I is highlighted in green in the phylogenetic trees (Figure 2); ** Group II is highlighted in red in the phylogenetic trees (Figure 2).

**Table 6:** Comparison of the population genetic parameters estimated for five coding regions of CSDaV isolates from symptomatic (Symp.) and asymptomatic (Asymp.) plants using the DnaSP and MEGA programs.

	Symptoms	Number of Sequences	π	θw	dN	dS	ω
**MT**	**Symp.**	13	0.01726	0.02117	0.007 ± 0.002	0.044 ± 0.011	0.159091
	**Asymp.**	11	0.00770	0.01402	0.002 ± 0.001	0.020 ± 0.005	0.100000
**MDR**	**Symp.**	13	0.03441	0.04143	0.026 ± 0.005	0.057 ± 0.011	0.456140
	**Asymp.**	11	0.02268	0.02601	0.014 ± 0.004	0.042 ± 0.009	0.333333
**He**	**Symp.**	2	0.00942	0.00942	0.002 ± 0.002	0.023 ± 0.012	0.086957
	**Asymp.**	4	0.00879	0.00924	0.003 ± 0.002	0.023 ± 0.008	0.130435
**RdRP**	**Symp.**	8	0.00856	0.00861	0.001 ± 0.001	0.026 ± 0.008	0.038462
	**Asymp.**	8	0.00787	0.00918	0.001 ± 0.001	0.024 ± 0.007	0.041667
**CP**	**Symp.**	9	0.00781	0.00912	0.003 ± 0.001	0.019 ± 0.008	0.157895
	**Asymp.**	11	0.00862	0.01317	0.004 ± 0.002	0.018 ± 0.006	0.222222

π: Nucleotide diversity, average pairwise nucleotide difference per site; θw: Mutation rate estimated from the total number of segregating sites; dN: The average number of pairwise differences per synonymous site; dS: The average number of pairwise differences per non-synonymous site. dS and dN were estimated by the Kumar method.

**Table 7:** Amino acid changes in five CSDaV genomic regions in isolates obtained from symptomatic citrus plants compared to isolates obtained from asymptomatic citrus plants.

Domain	Number of Amino Acid Changes	Total Number of Amino Acid	Position of Amino Acid Changes (Asymp→Symp)
**MT**	8	202	13 (I→T); 57 (P→Q); 113 (Q→R); 144 (L→V); 152 (S→*); 157 (R → K); 171 (A→V) and 199 (T→I)
**MDR**	10	209	13 (G→D); 14 (P→R); 22 (L→A); 29 (I→T); 103 (F→S); 109 (F→S); 110 (Q→P; 187 (S→L); 197 (H→R) and 209 (Q→R)
**He**	4	176	28 (V→A); 62 (L→P); 72 (T→I) and 120 (M→I)
**RdRP**	4	223	37 (P→L); 136 (A→V); 155 (N→ S) and 200 (L→P)
**CP**	1	120	55 (Q→R)

## Supplementary Materials

The following are available online at http://www.mdpi.com/1999-4915/8/12/330/s1, Figure S1: Graphical summary showing the conserved domains detected from conserved domain search using the CSDaV AY884005 reference sequence as query in the NCBI Conserved Domain Database (CDD), Figure S2: Graphical summary showing the conserved domains detected from conserved domain search using the CSDaV DQ185573 reference sequence as query in the NCBI Conserved Domain Database (CDD), Figure S3: Bootstrap majority rule (70%) consensus trees reconstructed by the neighbor joining method for five genomic regions of CSDaV isolates including the consensus sequences from transcriptome sequencing of the symptomatic and asymptomatic plants by using Illumina platform, Figure S4: Bootstrap majority rule (70%) consensus trees reconstructed by the neighbor joining method for five genomic regions of CSDaV isolates including possible recombinant isolates, field collected and reference sequences, Table S1: Description of domains detected from conserved domain search using the CSDaV AY884005 reference sequence as query. The interval and E-value of each identified domain are shown, Table S2: Description of domains detected from conserved domain search using the CSDaV DQ185573 reference sequence as query. The interval and E-value of each identified domain are shown.
