# Supplementary material for: Genetic Structure and Molecular Variability Analysis of Citrus sudden death-associated virus Isolates from Infected Plants Grown in Brazil
Source: Viruses. 2016 Dec 16;8(12):330. doi: 10.3390/v8120330 (PMC5192391; doi:10.3390/v8120330)
Supplement: Supplementary file 1 [file viruses-08-00330-s001.docx]

Supplementary Materials: Genetic Structure and Molecular Variability Analysis of *Citrus sudden death-associated virus* Isolates from Infected Plants Grown in Brazil

Emilyn Emy Matsumura, Helvécio Della Coletta-Filho, Silvia de Oliveira Dorta,
Shahideh Nouri and Marcos Antonio Machado


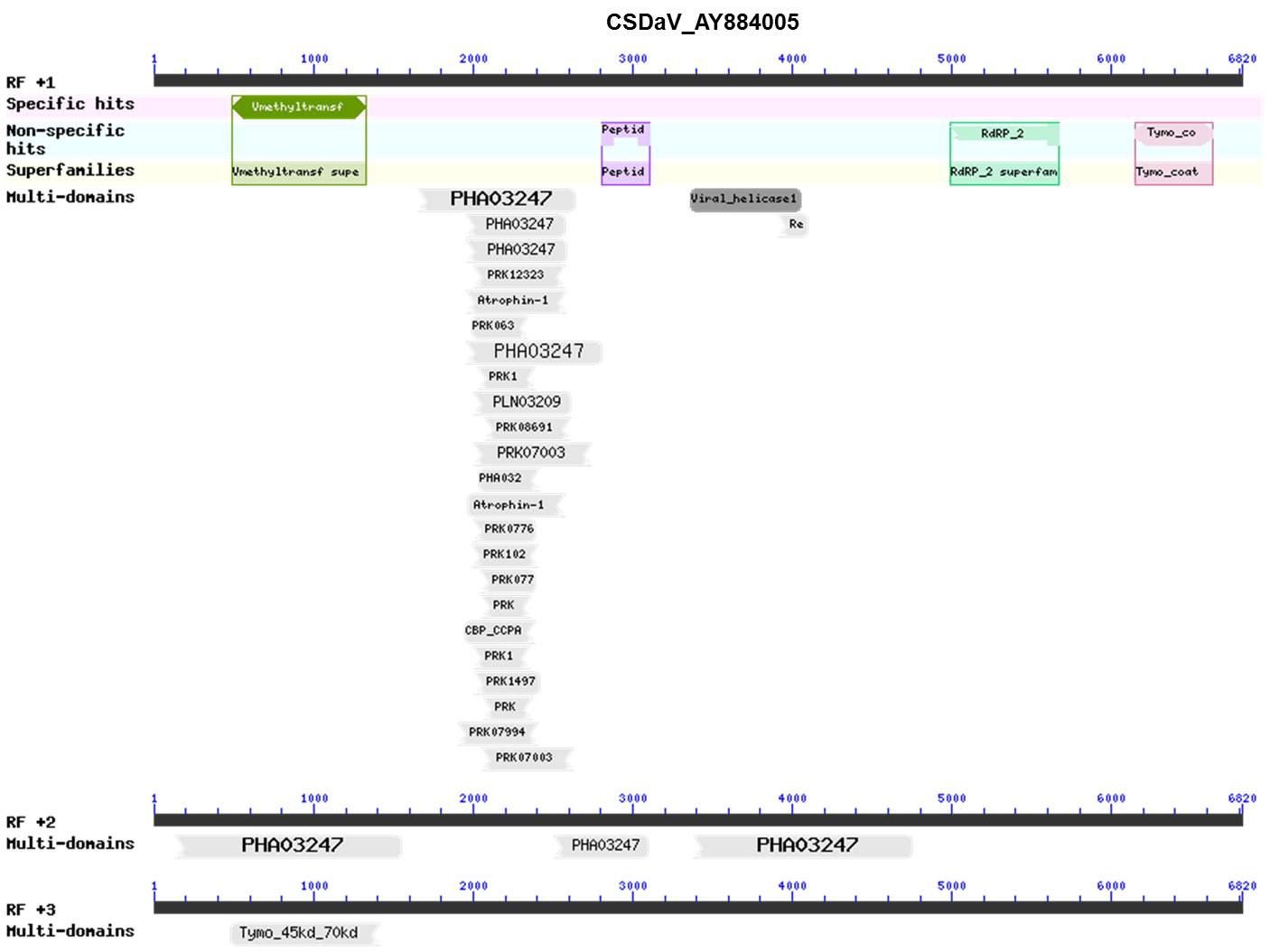


**Figure S1.** Graphical summary showing the conserved domains detected from conserved domain search using the CSDaV AY884005 reference sequence as query in the NCBI Conserved Domain Database (CDD).


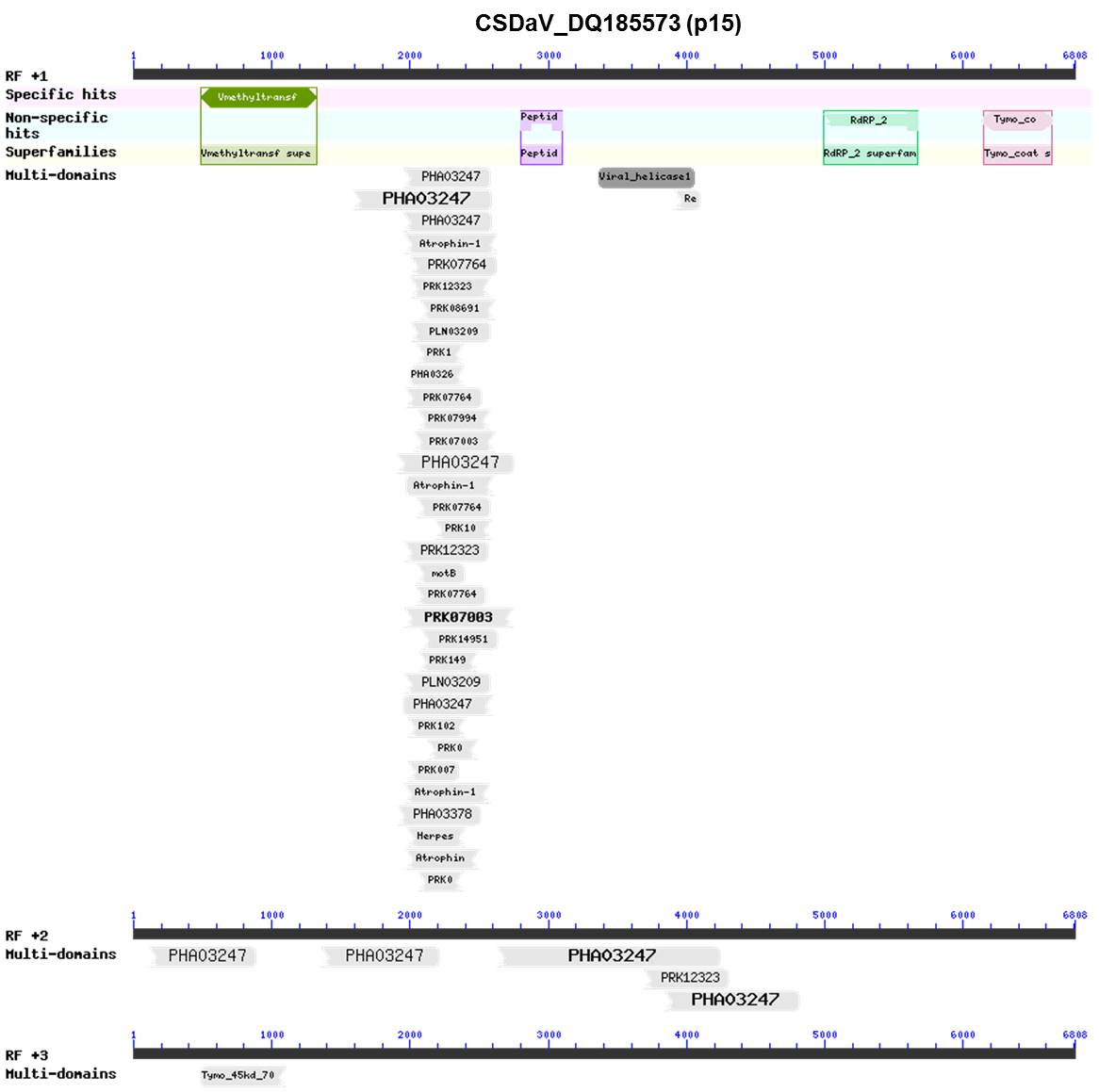


**Figure S2.** Graphical summary showing the conserved domains detected from conserved domain search using the CSDaV DQ185573 reference sequence as query in the NCBI Conserved Domain Database (CDD).


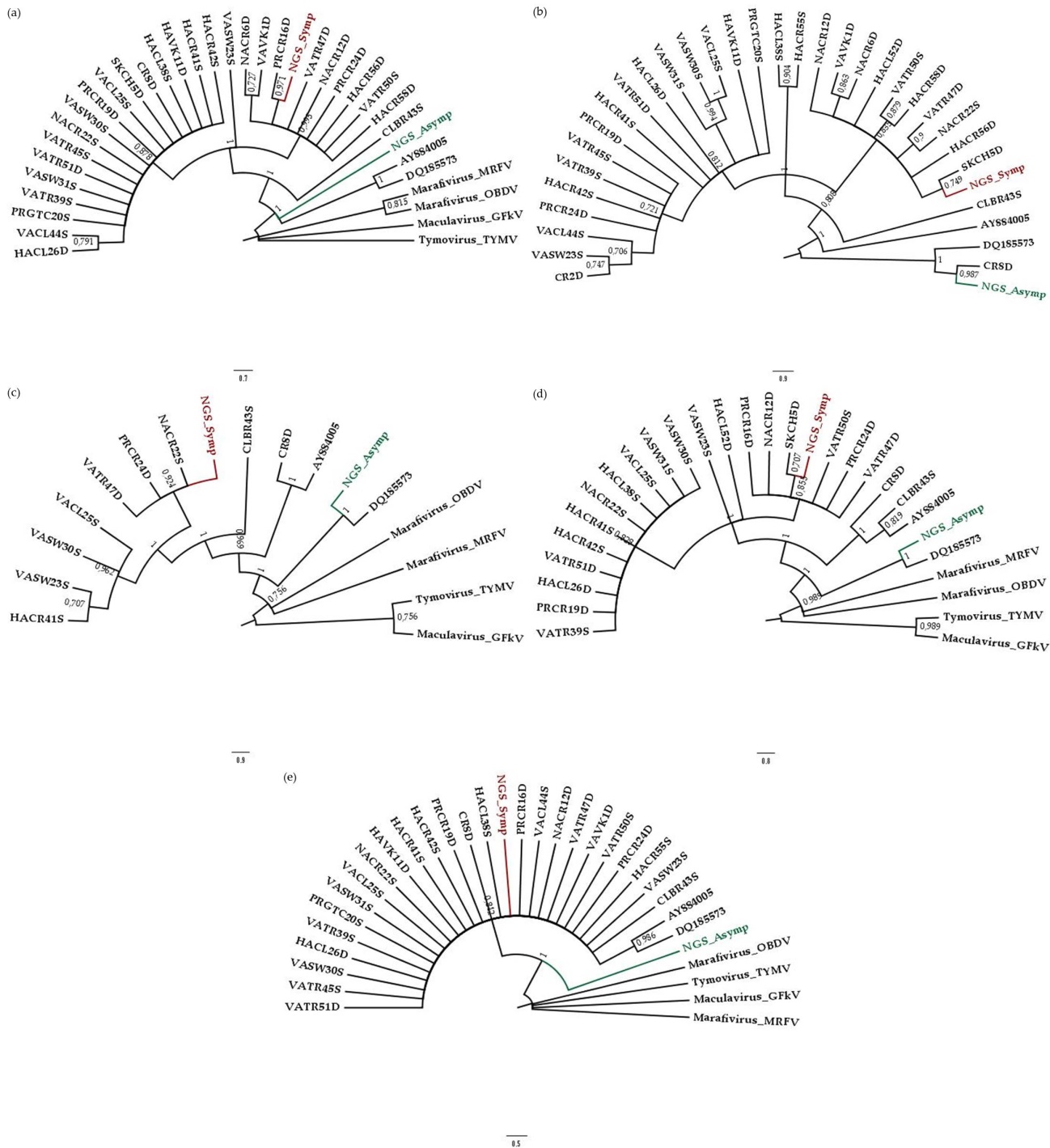


**Figure S3.** Bootstrap majority rule (70%) consensus trees reconstructed by the neighbor joining method for five genomic regions of CSDaV isolates including the consensus sequences from transcriptome sequencing of the symptomatic and asymptomatic plants by using Illumina platform. Bootstrap values are given above branches. (**a**) MT segment; (**b**) p60 segment; (**c**) He segment; (**d**) RdRP segment; (**e**) CP segment. CSDaV consensus sequences are differentiated by colors: From asymptomatic plants = green; from symptomatic plants = red.


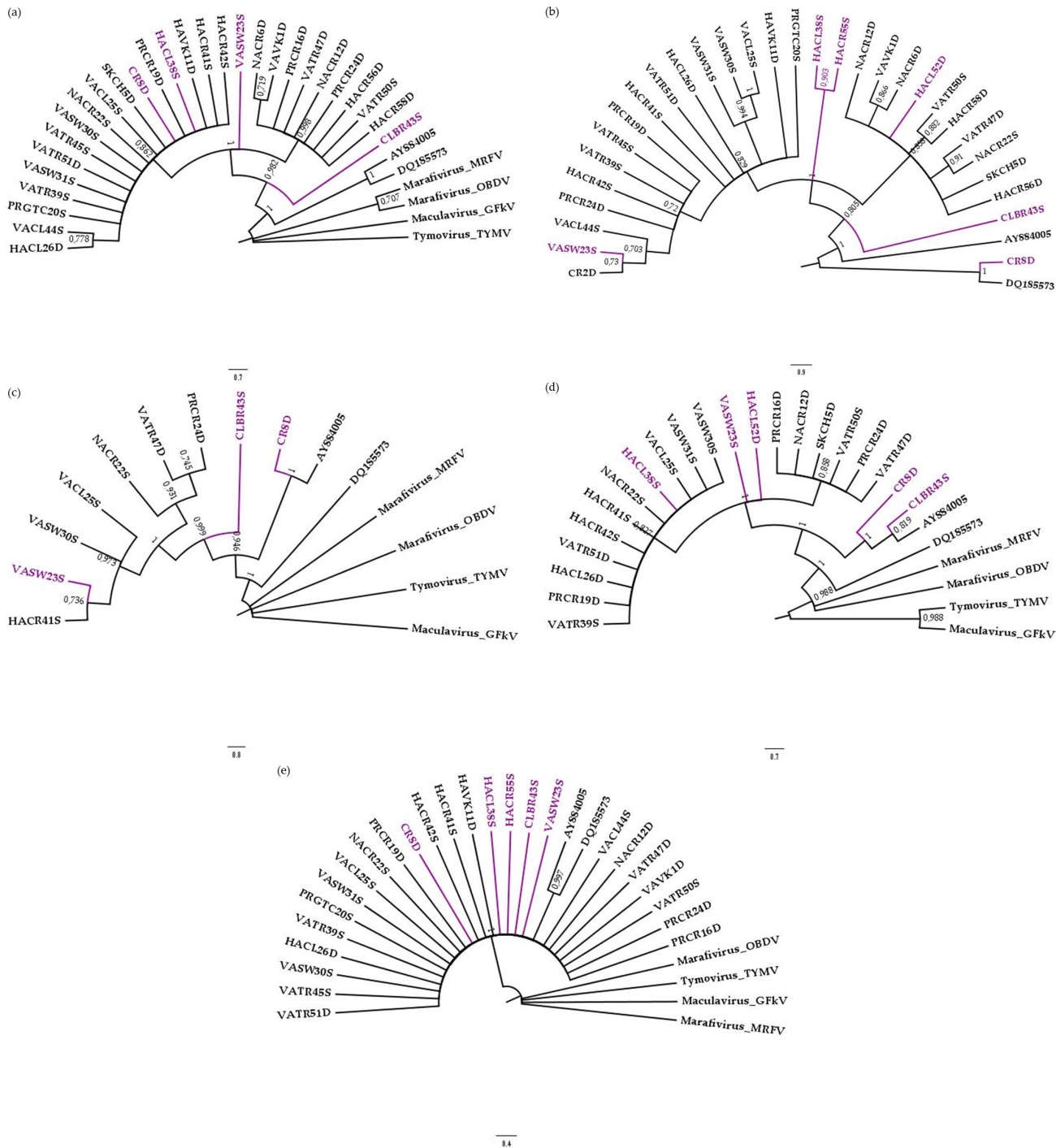


**Figure S4.** Bootstrap majority rule (70%) consensus trees reconstructed by the neighbor joining method for five genomic regions of CSDaV isolates including possible recombinant isolates, field collected and reference sequences. Bootstrap values are given above branches. (**a**) MT segment; (**b**) p60 segment; (**c**) He segment; (**d**) RdRP segment; (**e**) CP segment. The possible recombinant isolates are represented by purple color.

**Table S1.** Description of domains detected from conserved domain search using the CSDaV AY884005 reference sequence as query. The interval and E-value of each identified domain are shown.

| **Name** | **Accession** | **Description** | **Interval** | **E-Value** |
| --- | --- | --- | --- | --- |
| Vmethyltransf | pfam01660 | Viral methyltransferase; This RNA methyltransferase domain is found in a wide range of ssRNA ... | 484–1329 | 1.59e-77 |
| Peptidase_C21 | pfam05381 | Tymovirus endopeptidase; Corresponds to Merops family C21. The best-studied plant alpha-like ... | 2803–3102 | 7.52e-20 |
| RdRP_2 | pfam00978 | RNA dependent RNA polymerase; This family may represent an RNA dependent RNA polymerase. The ... | 4990–5670 | 4.65e-11 |
| Tymo_coat | pfam00983 | Tymovirus coat protein | 6148–6639 | 6.90e-11 |
| Viral_helicase1 | pfam01443 | Viral (Superfamily 1) RNA helicase; Helicase activity for this family has been demonstrated ... | 3361–4053 | 3.24e-50 |
| PHA03247 | PHA03247 | large tegument protein UL36; Provisional | 1651–2640 | 2.37e-08 |
| PHA03247 | PHA03247 | large tegument protein UL36; Provisional | 1960–2577 | 9.15e-08 |
| PHA03247 | PHA03247 | large tegument protein UL36; Provisional | 1954–2586 | 1.21e-06 |
| PRK12323 | PRK12323 | DNA polymerase III subunits gamma and tau; Provisional | 2002–2577 | 2.39e-06 |
| Atrophin-1 | pfam03154 | Atrophin-1 family; Atrophin-1 is the  protein product of the dentatorubral-pallidoluysian ... | 1957–2583 | 3.61e-06 |
| PRK06347 | PRK06347 | autolysin; Reviewed | 1987–2349 | 8.60e-06 |
| PHA03247 | PHA03247 | large tegument protein UL36; Provisional | 1954–2805 | 1.56e-05 |
| PRK14971 | PRK14971 | DNA polymerase III subunits gamma and tau; Provisional | 2029–2385 | 2.14e-05 |
| PLN03209 | PLN03209 | translocon at the inner envelope of chloroplast subunit 62; Provisional | 2002–2613 | 9.59e-05 |
| PRK08691 | PRK08691 | DNA polymerase III subunits gamma and tau; Validated | 2074–2613 | 1.17e-04 |
| PRK07003 | PRK07003 | DNA polymerase III subunits gamma and tau; Validated | 2002–2745 | 1.30e-04 |
| PHA03269 | PHA03269 | envelope glycoprotein C; Provisional | 2032–2415 | 1.31e-04 |
| Atrophin-1 | pfam03154 | Atrophin-1 family; Atrophin-1 is the  protein product of the dentatorubral-pallidoluysian ... | 1969–2574 | 1.42e-04 |
| PRK07764 | PRK07764 | DNA polymerase III subunits gamma and tau; Validated | 2002–2385 | 2.14e-04 |
| PRK10263 | PRK10263 | DNA translocase FtsK; Provisional | 1990–2406 | 3.64e-04 |
| PRK07764 | PRK07764 | DNA polymerase III subunits gamma and tau; Validated | 2041–2385 | 1.26e-03 |
| PRK07764 | PRK07764 | DNA polymerase III subunits gamma and tau; Validated | 2050–2361 | 2.32e-03 |
| CBP_CCPA | pfam17040 | Cellulose-complementing protein A; CBP_CCPA is a family of bacterial cellulose-complementing ... | 1945–2391 | 2.65e-03 |
| PRK14959 | PRK14959 | DNA polymerase III subunits gamma and tau; Provisional | 2002–2358 | 3.48e-03 |
| PRK14971 | PRK14971 | DNA polymerase III subunits gamma and tau; Provisional | 2005–2415 | 4.20e-03 |
| PRK07764 | PRK07764 | DNA polymerase III subunits gamma and tau; Validated | 2059–2370 | 4.31e-03 |

**Table S1.** *Cont.*

| **Name** | **Accession** | **Description** | **Interval** | **E-Value** |
| --- | --- | --- | --- | --- |
| PRK07994 | PRK07994 | DNA polymerase III subunits gamma and tau; Validated | 1900–2409 | 4.31e-03 |
| PRK07003 | PRK07003 | DNA polymerase III subunits gamma and tau; Validated | 2056–2628 | 4.41e-03 |
| RecD | COG0507 | ATP-dependent exoDNAse (exonuclease V), alpha subunit, helicase superfamily I Replication, ... | 3910–4098 | 7.98e-03 |
| PHA03247 | PHA03247 | large tegument protein UL36; Provisional | 134–1546 | 2.47e-05 |
| PHA03247 | PHA03247 | large tegument protein UL36; Provisional | 3377–4753 | 3.42e-05 |
| PHA03247 | PHA03247 | large tegument protein UL36; Provisional | 2504–3097 | 6.19e-04 |
| Tymo_45kd_70kd | pfam03251 | Tymovirus 45/70Kd protein; Tymoviruses are single stranded RNA viruses. This family includes a ... | 483–1418 | 4.49e-12 |

**Table S2.** Description of domains detected from conserved domain search using the CSDaV DQ185573 reference sequence as query. The interval and E-value of each identified domain are shown.

| **Name** | **Accession** | **Description** | **Interval** | **E-value** |
| --- | --- | --- | --- | --- |
| Vmethyltransf | pfam01660 | Viral methyltransferase; This RNA methyltransferase domain is found in a wide range of ssRNA ... | 484–1329 | 1.16e-76 |
| Peptidase_C21 | pfam05381 | Tymovirus endopeptidase; Corresponds to Merops family C21. The best-studied plant alpha-like ... | 2803–3102 | 3.70e-19 |
| RdRP_2 | pfam00978 | RNA dependent RNA polymerase; This family may represent an RNA dependent RNA polymerase. The ... | 4993–5673 | 4.40e-11 |
| Tymo_coat | pfam00983 | Tymovirus coat protein | 6151–6642 | 6.88e-11 |
| Viral_helicase1 | pfam01443 | Viral (Superfamily 1) RNA helicase; Helicase activity for this family has been demonstrated ... | 3361–4053 | 2.10e-49 |
| PHA03247 | PHA03247 | large tegument protein UL36; Provisional | 1951–2577 | 3.29e-09 |
| PHA03247 | PHA03247 | large tegument protein UL36; Provisional | 1594–2592 | 1.03e-07 |
| PHA03247 | PHA03247 | large tegument protein UL36; Provisional | 1954–2586 | 2.15e-07 |
| Atrophin-1 | pfam03154 | Atrophin-1 family; Atrophin-1 is the protein product of the dentatorubral-pallidoluysian ... | 1957–2628 | 4.51e-07 |
| PRK07764 | PRK07764 | DNA polymerase III subunits gamma and tau; Validated | 2002–2622 | 8.40e-07 |
| PRK12323 | PRK12323 | DNA polymerase III subunits gamma and tau; Provisional | 2002–2577 | 2.01e-06 |
| PRK08691 | PRK08691 | DNA polymerase III subunits gamma and tau; Validated | 2074–2613 | 1.61e-05 |
| PLN03209 | PLN03209 | translocon at the inner envelope of chloroplast subunit 62; Provisional | 2002–2577 | 2.41e-05 |
| PRK14971 | PRK14971 | DNA polymerase III subunits gamma and tau; Provisional | 2047–2385 | 2.55e-05 |
| PHA03269 | PHA03269 | envelope glycoprotein C; Provisional | 2008–2397 | 4.43e-05 |
| PRK07764 | PRK07764 | DNA polymerase III subunits gamma and tau; Validated | 1969–2514 | 5.84e-05 |
| PRK07994 | PRK07994 | DNA polymerase III subunits gamma and tau; Validated | 2056–2580 | 7.54e-05 |
| PRK07003 | PRK07003 | DNA polymerase III subunits gamma and tau; Validated | 2035–2613 | 9.69e-05 |
| PHA03247 | PHA03247 | large tegument protein UL36; Provisional | 1906–2745 | 1.41e-04 |
| Atrophin-1 | pfam03154 | Atrophin-1 family; Atrophin-1 is the protein product of the dentatorubral-pallidoluysian ... | 1969–2604 | 1.89e-04 |
| PRK07764 | PRK07764 | DNA polymerase III subunits gamma and tau; Validated | 2050–2577 | 4.09e-04 |
| PRK10263 | PRK10263 | DNA translocase FtsK; Provisional | 2185–2589 | 5.53e-04 |
| PRK12323 | PRK12323 | DNA polymerase III subunits gamma and tau; Provisional | 1957–2559 | 6.46e-04 |
| motB | PRK12799 | flagellar motor protein MotB; Reviewed | 2050–2391 | 7.16e-04 |
| PRK07764 | PRK07764 | DNA polymerase III subunits gamma and tau; Validated | 2041–2547 | 7.39e-04 |
| PRK07003 | PRK07003 | DNA polymerase III subunits gamma and tau; Validated | 1957–2745 | 1.00e-03 |

**Table S2.** *Cont.*

| **Name** | **Accession** | **Description** | **Interval** | **E-value** |
| --- | --- | --- | --- | --- |
| RecD | COG0507 | ATP-dependent exoDNAse (exonuclease V), alpha subunit, helicase superfamily I [Replication, ... | 3910–4098 | 1.81e-03 |
| PRK14951 | PRK14951 | DNA polymerase III subunits gamma and tau; Provisional | 2083–2634 | 2.52e-03 |
| PRK14959 | PRK14959 | DNA polymerase III subunits gamma and tau; Provisional | 2071–2490 | 2.79e-03 |
| PLN03209 | PLN03209 | translocon at the inner envelope of chloroplast subunit 62; Provisional | 1957–2583 | 3.79e-03 |
| PHA03247 | PHA03247 | large tegument protein UL36; Provisional | 1951–2595 | 4.08e-03 |
| PRK10263 | PRK10263 | DNA translocase FtsK; Provisional | 1990–2406 | 4.31e-03 |
| PRK07764 | PRK07764 | DNA polymerase III subunits gamma and tau; Validated | 2125–2490 | 4.88e-03 |
| PRK00708 | PRK00708 | sec-independent translocase; Provisional | 1990–2349 | 5.09e-03 |
| Atrophin-1 | pfam03154 | Atrophin-1 family; Atrophin-1 is the protein product of the dentatorubral-pallidoluysian ... | 1960–2583 | 5.98e-03 |
| PHA03378 | PHA03378 | EBNA-3B; Provisional | 1906–2505 | 7.08e-03 |
| Herpes_BLLF1 | pfam05109 | Herpes virus major outer envelope glycoprotein (BLLF1); This family consists of the BLLF1 ... | 1975–2400 | 7.60e-03 |
| Atrophin-1 | pfam03154 | Atrophin-1 family; Atrophin-1 is the protein product of the dentatorubral-pallidoluysian ... | 1969–2499 | 8.38e-03 |
| PRK07764 | PRK07764 | DNA polymerase III subunits gamma and tau; Validated | 2062–2388 | 9.60e-03 |
| PHA03247 | PHA03247 | large tegument protein UL36; Provisional | 2630–4237 | 5.22e-04 |
| PRK12323 | PRK12323 | DNA polymerase III subunits gamma and tau; Provisional | 3695–4297 | 9.15e-04 |
| PHA03247 | PHA03247 | large tegument protein UL36; Provisional | 3839–4813 | 1.17e-03 |
| PHA03247 | PHA03247 | large tegument protein UL36; Provisional | 116–886 | 4.63e-03 |
| PHA03247 | PHA03247 | large tegument protein UL36; Provisional | 1358–2206 | 7.60e-03 |
| Tymo_45kd_70kd | pfam03251 | Tymovirus 45/70Kd protein; Tymoviruses are single stranded RNA viruses. This family includes a ... | 483–1106 | 3.79e-07 |

© 2016 by the authors; licensee MDPI, Basel, Switzerland. This article is an open access article distributed under the terms and conditions of the Creative Commons by Attribution (CC-BY) license (http://creativecommons.org/licenses/by/4.0/).
